# Tumoral and stromal expression of MMP-2, MMP-9, MMP-14, TIMP-1, TIMP-2, and VEGF-A in cervical cancer patient survival: a competing risk analysis

**DOI:** 10.1186/s12885-020-07150-3

**Published:** 2020-07-15

**Authors:** Jordana Maria Azevedo Martins, Silvia Helena Rabelo-Santos, Maria Cristina do Amaral Westin, Luiz Carlos Zeferino

**Affiliations:** 1 Department of Gynecology and Obstetrics, School of Medical Sciences, State University of Campinas, UNICAMP, Tessalia Vieira de Camargo Street, 126, Campinas, Sao Paulo 13083-887 Brazil; 2 School of Pharmacy, Federal University of Goias, 240 Street, Leste Universitario, Goiania, Goias 74605-170 Brazil; 3 Laboratory of Cytopathology, Women’s Health Hospital Professor Jose Aristodemo Pinotti – (CAISM), University of Campinas (UNICAMP), Campinas, Sao Paulo 13083-881 Brazil

**Keywords:** Uterine cervical neoplasms, Matrix metalloproteinase 2, Matrix metalloproteinase 9, Tissue inhibitor of Metalloproteinase-2, Tumor microenvironment, Stroma, Prognosis

## Abstract

**Background:**

Expression of matrix metalloproteases 2, 9 and 14 (MMP-2, MMP-9, MMP-14), tissue inhibitors of metalloprotease 1 and 2 (TIMP-1, TIMP-2) and vascular endothelial growth factor A (VEGF-A) is involved in tumor invasion and metastasis via extracellular matrix degradation and angiogenesis. This study aimed to assess whether the expression of MMP-2, MMP-9, MMP-14, TIMP-1, and TIMP-2 in tumors and in the adjacent stroma is associated with cervical cancer prognosis.

**Methods:**

This study analyzed a retrospective cohort of 64 patients. Protein expression was previously obtained by immunohistochemistry from biopsies containing both tumor and stroma. The expression and percentage of stained cells were categorized as high or low according to the cutoff points by using ROC curves. The follow-up data was collected from diagnosis to the last clinical visit. Clinical status categorized as alive without disease, alive with disease, death due to other causes, and death from the disease. The relative risk of death from the disease was evaluated according to the proteins expression using a cause-specific Cox regression model with a 95% confidence interval (95%CI). For the significant associations (*p* < 0.05), survival curves of patients with low and high expression were plotted for the competing risk survival curve analyses.

**Results:**

High expression levels of stromal MMP-2 (RR; 95%CI: 3.91; 1.17–13.02) and stromal TIMP-2 (RR, 95%CI: 8.67; 1.15–65.27) were associated with a greater relative risk of death from the disease and with lower survival (*p* = 0.03; *p* = 0.04) than lower expression levels. Low expression levels of stromal MMP-9 (RR, 95%CI: 0.19; 0.05–0.65) and tumoral MMP-9 (HR, 95%CI: 0.19; 0.04–0.90) were protective factors against death from the disease and were associated with poorer survival.

**Conclusions:**

High expression levels of MMP-2 and TIMP-2 in the stroma were significantly associated with poor survival in cervical cancer patients. High expression of MMP-9 was associated with a favorable cervical cancer prognosis.

## Background

High-risk human papillomavirus (HPV) is essential for the development of cervical intraepithelial neoplasias and invasive cervical carcinoma [1, 2]. Invasion takes place when tumor cells start to cross-talk with stromal cells, leading to cooperative enzymatic degradation of the basement membrane (BM) and the subjacent extracellular matrix (ECM), which allows the tumor to access vascularization to grow and metastasize [3–5].

The BM and ECM contain several proteins, such as collagens, fibronectin, and laminin [6], and so, there are several proteolytic enzymes that are involved in invasion [7], including matrix metalloproteinases (MMPs). MMPs are essential in the degradation process and also promote cellular migration, regulate growth factors and cytokines, influence apoptosis and collaborate in neovascularization. The expression of these proteins is regulated by their physiological inhibitors: tissue inhibitors of metalloproteinases (TIMPs). The expression levels of these proteins in tumoral, stromal, and inflammatory cells can be considered potential tumoral microenvironment invasion markers [8, 9].

**Fig. 1:**
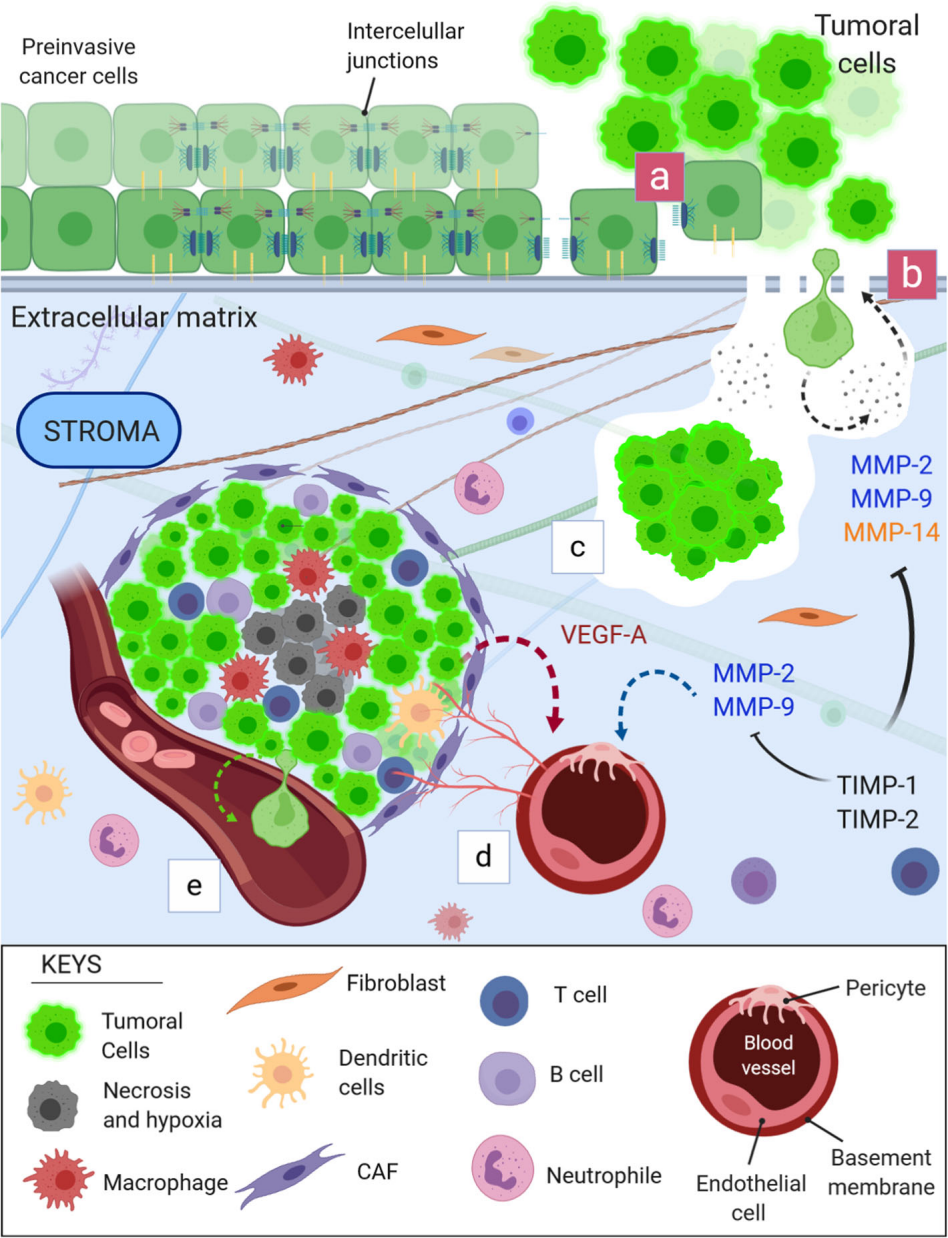
MMP-2, MMP-9, MMP-14, TIMP-1, TIMP-2, and VEGF-A in the process of tumor invasion, tumor progression, angiogenesis and metastasis. a) The invasion process begins with the disruption of intracellular junctions of carcinoma cells in situ and their adherence to the basement membrane. The basement membrane is then destabilized, opening spaces that allow enzymatic action. b) Tumor cells continue the process by secreting proteolytic enzymes, such as MMP-2, MMP-9, and MMP-14, which degrade collagen and other proteins present in the basement membrane. BM degradation allows tumoral cell invasion. At the same time, stromal cells, now reactive, start to produce more extracellular matrix and stiffen collagen fibers, which facilitates cell motility. c) Once invaded, the tumor cells continue to secrete enzymes to degrade the surrounding ECM, which allows migration and tumoral enlargement. d) The tumor cells interact with the stromal cells and the extracellular matrix and form the tumor microenvironment. As the tumor grows, the innermost tumoral zone is deprived of oxygen and nutrients, developing a hypoxic and necrotic core. Tumor cells start to secrete VEGF-A in abundance to stimulate the formation of new blood vessels. MMPs, in particular MMP-9 and MMP-2, degrade the basement membrane of adjacent vessels, exposing VEGF-A receptors expressed on pericytes and endothelial cells. VEGF-A initiates the angiogenesis process for tumor survival and progression. e) Due to the rupture of the basement membrane for the formation of new vessels, similar to what initially occurs, the opening of spaces and the continuous action of the MMPs, there is a propensity for distant metastasis, since tumor cells can disseminate into the bloodstream. TIMP-1 and TIMP-2 directly inhibit MMP-2, MMP-9, and MMP-14 and are therefore related to the regulation of their activity in tissues

There are at least 23 MMPs and four types of TIMPs expressed in humans, but MMP-2, MMP-9, MMP-14, TIMP-1, and TIMP-2 are often associated with the tumor invasion process [10, 11]. MMP-2 and MMP-9 present high proteolytic action against type IV collagen, which is abundant in BM [12, 13]. MMP-14, on the other hand, degrades other basement membrane components, such as collagen type I [14]. TIMP-1, associated with MMPs, has been described as a predictive factor of survival and might predict recurrence in certain types of cancer [15, 16]. In contrast, TIMP-2 is the only TIMP protein that can inhibit the mitogenic response independently of MMP [17]. MMP-2, − 9 and, − 14 also interact with vascular endothelial growth factor (VEGF), stimulating angiogenesis, another process to enhance tumoral growth and development [18]. VEGF-A, referred only to as VEGF, is strongly associated with worse survival and is already a therapeutic target. Bevacizumab is a humanized monoclonal anti-VEGF antibody that can increase the overall survival of women with advanced cervical cancer [19]. Figure 1 illustrates the performance of the six aforementioned proteins in the context of cancer invasion, migration, progression and metastasis.

The treatment for invasive cervical cancer is surgery for patients in earlier stages and concurrent chemoradiation in patients with stage IB3 or more advanced cancer [20]. However, recurrence rates are still high for patients who have advanced-stage tumors. Cisplatin-based chemotherapy, added to standard radiation therapy, reduces recurrence risk and disease-specific death in 50% of patients, but the other half of the patients still present a poor prognosis [21]. To improve these clinical outcomes, identifying more aggressive cases could be useful for testing adjuvant therapy or more aggressive primary treatments. In this way, biomarkers could be helpful to identify these more aggressive cases.

This study aimed to assess whether the expression of MMP-2, MMP-9, MMP-14, TIMP-1, and TIMP-2 in tumors and in the adjacent stroma is associated with cervical cancer prognosis. If there is a positive association, these markers could represent potential therapeutic targets for improving the clinical outcome of cervical cancer patients. VEGF-A was included in this analysis because it interacts with MMPs in the angiogenesis and metastasis process and because it is already known to be associated with a poor prognosis.

## Methods

### Case selection and ethics

This is a cohort study that included women treated at the Woman’s Hospital Professor José Aristodemo Pinotti - CAISM, State University of Campinas (UNICAMP). They were diagnosed between January 2005 and December 2008, and their last clinical information was collected up to June 2018. The first selection step was to identify the reports with the diagnosis of invasive squamous cell carcinoma of the cervix associated with cervical intraepithelial neoplasia (CIN) III and reports with only invasive squamous cell carcinoma. After that, the formalin-fixed paraffin-embedded tissues were retrieved, and new slides stained by hematoxylin and eosin were prepared to identify the cases with tumors and adjacent stroma in the available specimens. Stage I patients underwent conization so they had larger specimens: more likely to find tumor and stroma tissues represented. Patients in stages II to IV frequently had a diagnosis based on smaller specimens derived from punch biopsies, and most of them did not obtain enough stromal tissue for the immunohistochemistry assay.

**Fig. 2:**
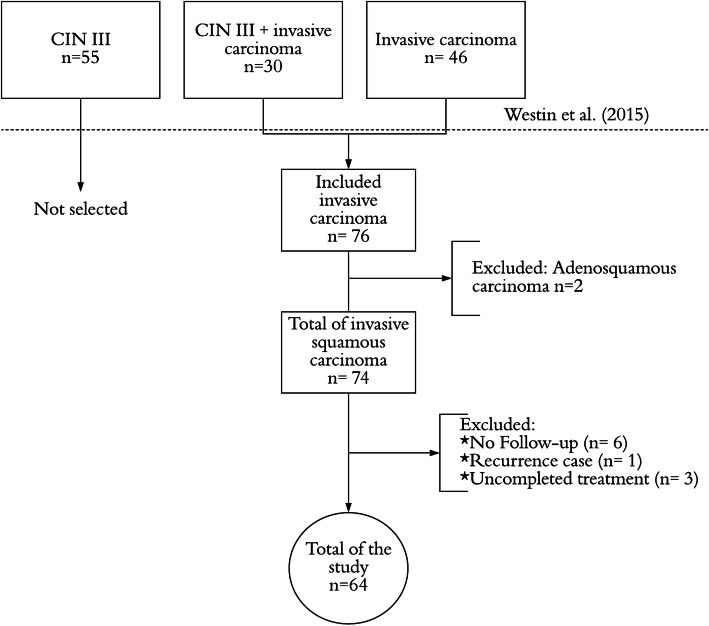
Flow chart of patient selection

Seventy-six women fit the inclusion criteria: invasive carcinoma with stromal and tumoral tissues in the same specimen. Two of them were excluded because they were not squamous carcinomas, and the other ten patients were excluded because one woman had a recurrent disease; three women that did not adhere to the proposed treatment; and six that had no follow-up. Therefore, 64 patients were selected for this study (Fig. 2).

The initial case selection and immunohistochemistry assay were approved by the Research Ethics Committee of State University of Campinas (CAAE: 0614.0.146.000–07). The actual study was performed following the Declaration of Helsinki [22], and it was approved by the Research Ethics Committee of State University of Campinas (CAAE: 83156117.8.0000.5404). Considering that the study was retrospective, written consent forms were requested only for those patients who were still being treated at the hospital. All the clinical data were collected directly from the medical files, and the patients’ identities were protected.

### Clinical procedures

The diagnosis, staging, and treatment followed the International Federation of Gynecology and Obstetrics (FIGO) recommendations [23, 24]. The standard treatment was either a simple or radical hysterectomy for stages IA1 to IB1 and a combination of external beam radiotherapy with cisplatin and high-dose-rate brachytherapy for stages IB2 to IVA. The standard clinical follow-up interval was between 4 and 6 months for the first five years and then every 12 months thereafter. At every follow-up evaluation, the women passed through physical and gynecological examination and had cytological samples collected. Imaging evaluations were performed according to the findings of the clinical evaluation.

### Immunohistochemistry assay

The immunohistochemical analyses for MMP-2, MMP-9, MMP-14, TIMP-1, TIMP-2, and VEGF-A were performed as described previously by Westin et al. through hot spots areas [25, 26]. Total cells and immunostained cells (stromal and tumor cells separately) were counted by two observers using morphometric software (Image-Pro Plus®, version 6.3, Olympus). The methodology for interpretation of the immunostained sections consisted of the following steps:

- 1 – Representative regions were identified from the lesions selected: the hot spots. The selection criteria for these representative areas were based on image sharpness, areas of higher intensity of cellular immunoreaction, positive staining (any intensity of immunostaining), and a similar proportion of stromal and tumor regions in the same picture, excluding necrotic regions. Stromal regions with a high concentration of inflammatory cells were excluded.
- 2 – Images of these regions were captured under a magnification of 400X.
- 3 - One photograph per lesion and its underlying stroma was selected for quantitative and qualitative analysis.
- 4 - Total cells and immunostained cells (stromal and tumor cells separately) were counted by two observers using morphometric software (Image-Pro Plus®, version 6.3, Olympus). To determine the percentage of immunostained cells, at least 1000 tumor cells and stromal cells per case were counted.

### Data collection

This study used the MMP-2, MMP-9, MMP-14, TIMP-1, TIMP-2 and VEGF-A analyses that were available in Westin’s immunohistochemistry database. The database contains the total number and the percentage of stained cells in the tumor and stroma. The following clinical data were obtained from the medical files: diagnosis date, age at diagnosis, histological type, histopathological grade (classified as G1: well-differentiated, G2: moderately differentiated, G3: poorly or undifferentiated and GX: grade cannot be assessed), tumor stage (I, II, III or IV), start and end dates of the treatment, recurrence date (if applicable), death date, and death cause (if applicable). The deaths were classified according to the International Statistical Classification of Diseases and Related Health Problems (ICD) C53 - Malignant Neoplasm of Cervix Uteri. The follow-up data were collected up to June 2018, and patient clinical status was categorized as alive without disease, alive with disease, death due to other causes, and death from the disease.

### Statistical analysis

#### Protein expression categorization

**Fig. 3:**
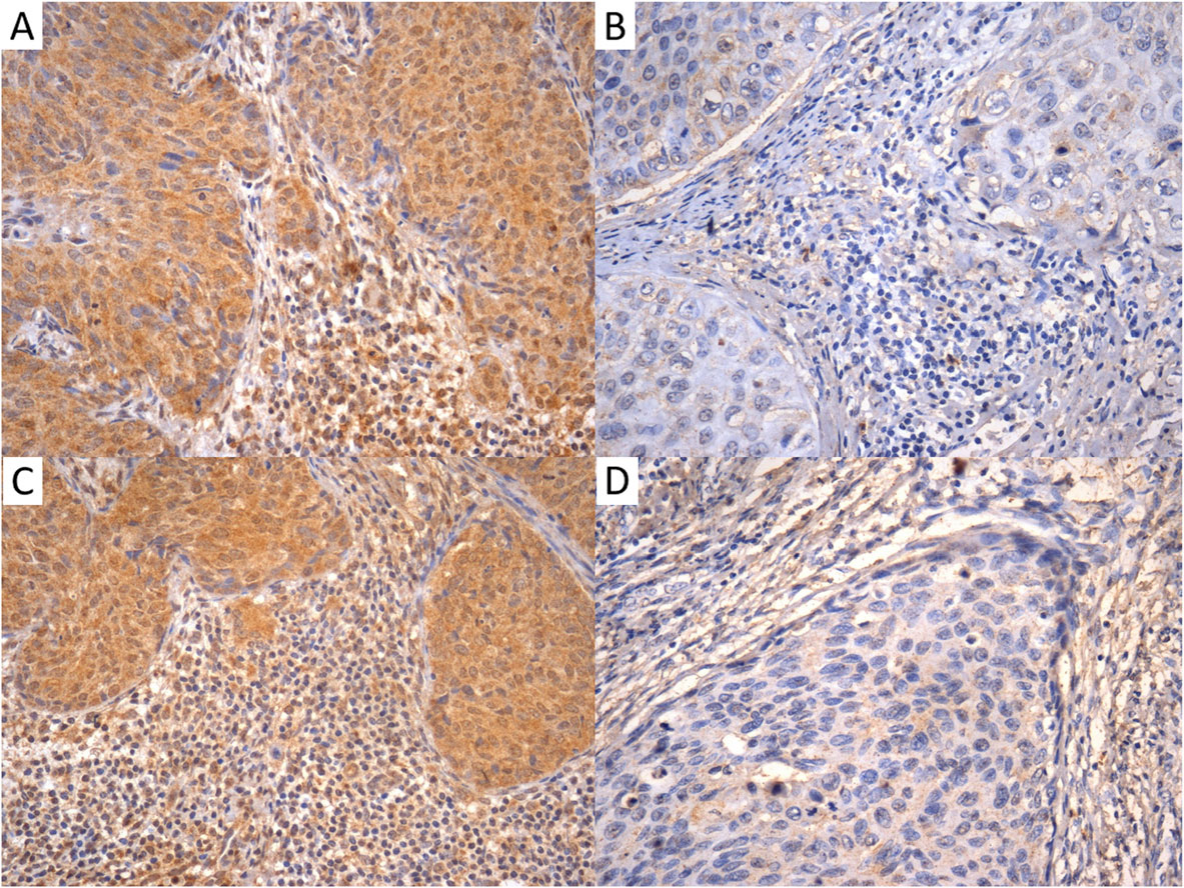
Percentages of stromal and tumoral cells and their categorization as high or low expression according to ROC cutoff points. A: MMP-2. High expression in stromal cells (91.0%) and tumor cells (89.7%); B: MMP-2. Low expression in stromal cells (46.7%) and tumor cells (36.4%); C: MMP-14. High expression in stromal cells (97.1%) and tumor cells (85.5%), D: MMP-14. Low expression in stromal cells (12.1%) and tumor cells (20.4%)

For analysis purposes, the expression of each protein in stromal and tumoral tissues was categorized by a cutoff point, determined by ROC curves, that used death from the disease as a reference. Therefore, there were twelve cutoff points (six proteins in tumoral and stromal tissue) (Table 1). The categories “high expression” and “low expression” were used, respectively, for percentages above and below their respective cutoff point. Figure 3 shows two fields of MMP-2 and two fields of MMP-14 to illustrate high and low expressions.

**Table 1:** Cutoff* points for the protein expressions**

Proteins	Expression (%)	
	High	Low
MMP-2 tumor	≥29.8	< 29.8
MMP-2 stroma	≥75.5	< 75.5
MMP-9 tumor	≥44.5	< 44.5
MMP-9 stroma	≥26.5	< 26.5
MMP-14 tumor	≥82.2	< 82.2
MMP-14 stroma	≥66.7	< 66.7
TIMP-1 tumor	≥92.0	< 92.0
TIMP-1 stroma	≥88.1	< 88.1
TIMP-2 tumor	≥44.4	< 44.4
TIMP-2 stroma	≥60.6	< 60.6
VEGF-A tumor	≥64.9	< 64.9
VEGF-A stroma	≥49.7	< 49.7

*Cutoff points defined by ROC curves, 95% confidence interval. **Determined by the percentage of stained cells

#### Cause-specific cox regression model

A regression model of competitive risk survival was used considering two events of interest: death due to other causes and death from the disease. Since it is a cause-specific survival model, the cases with death due to other causes status were censored but not excluded. The relative risk (RR) of death from cervical cancer was considered statistically significant at a *p*-value of less than 0.05, with a 95% confidence interval (95%CI). The competing risk survival analyses were performed with competing low and high expression curves plotted for the significant ones using the same regression model. The software R version 3.6.0 was used to perform the analyses.

## Results

### Patient characteristics

The patient characteristics are described in Table 2. Stage I was the most frequent (71.9%), followed by stage III (20.3%). Among the histopathological grades, G2 was the most frequent (49.2%), followed by G3 (30.2%). At the last clinical visit, 64.1% of the patients were alive without disease, 15.6% had died due to other causes and 18.8% had died due to cervical cancer. Among this 18.8%, which represents 12 patients, 10 died in advanced stages (stage II-IV), and only 2 died in stage I.

**Table 2:** Pathological and clinical characteristics of the patients

Total number of patients		64	
Mean age (min-max) ^a^		47 (22–94)	
Median age ^a^		44	
Mean follow-up (min-max) ^b^		5.6 (0.5–12.6)	
Recurrences		8	
		n	%
FIGO* stage	I	46	71.9
	II	3	4.7
	III	13	20.3
	IV	2	3.1
Histopathological grade	G1	13	20.6
	G2	31	49.2
	G3	19	30.2
	(Missing = 1)**		
Clinical status	Alive without disease	41	64.1
	Alive with disease	1	1.6
	Death due to other causes	10	15.6
	Death from the disease	12	18.8

^a^ complete years of age; ^b^ measured in years; *International Federation of Gynecology and Obstetrics; **the original report did not contain this information, and it was not possible to find the slide for reanalysis

### Competing risk survival analyses

Cause-specific Cox regression analyses for competing risk survival (CRS) according to age, tumor stage, histopathological grade, and protein expression are shown in Table 3. Women older than 44 years old (RR; 95%CI: 9.46; 1.21–74.05) and women with advanced stage (II-IV) disease (RR; 95%CI: 15.17; 0.01–0.29) had a higher risk of death from the disease.

**Table 3:** Competing risk survival analysis according to clinical characteristics, tumor stage and protein expression*

Variables ^**∆**^	Cause-specific Cox regression		
Univariate analysis (***n*** = 64)	RRª	95%CI	p-value
Age ^b^ (> 44 vs. ≤44 years)	9.46	1.21–74.05	0.03
Tumor stage (II-IV vs. I)	15.17	0.68–6.70	< 0.001
Histopathological grade (G2-G3 vs. G1)	0.51	0.01–0.29	0.19
MMP-2 tumor	–	–	< 0.000^ß^
MMP-2 stroma	3.91	1.17–13.02	0.03
MMP-9 tumor	0.19	0.04–0.90	0.04
MMP-9 stroma	0.19	0.05–0.65	0.01
MMP-14 tumor	3.57	0.43–29.90	0.24
MMP-14 stroma	7.11	0.88–57.58	0.07
TIMP-1 tumor	0.45	0.06–3.56	0.45
TIMP-1 stroma	2.30	0.67–7.89	0.19
TIMP-2 tumor	3.19	0.90–11.55	0.07
TIMP-2 stroma	8.67	1.15–65.27	0.04
VEGF-A tumor	3.23	0.90–11.55	0.07
VEGF-A stroma	3.77	0.80–117.73	0.09

*The expression was analyzed based on the percentage of stained cells and categorized by the ROC curve; ^**∆**^The first category is the reference ^a^RR = relative risk. ^ß^ All the women showing higher expressions of tumoral MMP-2 entered died from the disease before the analysis began, so the relative risk could not be assessed

The cause-specific death risk was greater in those cases with high expression of MMP-2 in the stroma (RR; 95%CI: 3.91; 1.17–13.02) and TIMP-2 in the stroma (HR; 95%CI: 8.67; 1.15–65.27). In contrast, high expression of MMP-9 in the stroma (RR; 95%CI: 0.19; 0.05–0.65) and in the tumor (HR; 95%CI: 0.19; 0.04–0.90) had a protective effect on cervical cancer prognosis (Table 3).

All the women showing high expression of tumoral MMP-2 died from the disease before the analysis began. For this reason, there was no way to determine the difference between patients with high and low expression of this protein; hence, this association lacks a tangible relative risk. The association was statistically significant because all the women with higher MMP-2 in the tumor died from the disease, but there was no comparison with low expression, so the competing risk survival curves could not be assessed.

The competing risk survival curves were plotted for the five significant associations in the cause-specific regression model; however, as previously explained, the tumoral MMP-2 variable had a complete separation between high and low expression levels, so survival analysis was not possible for this association.

**Fig. 4:**
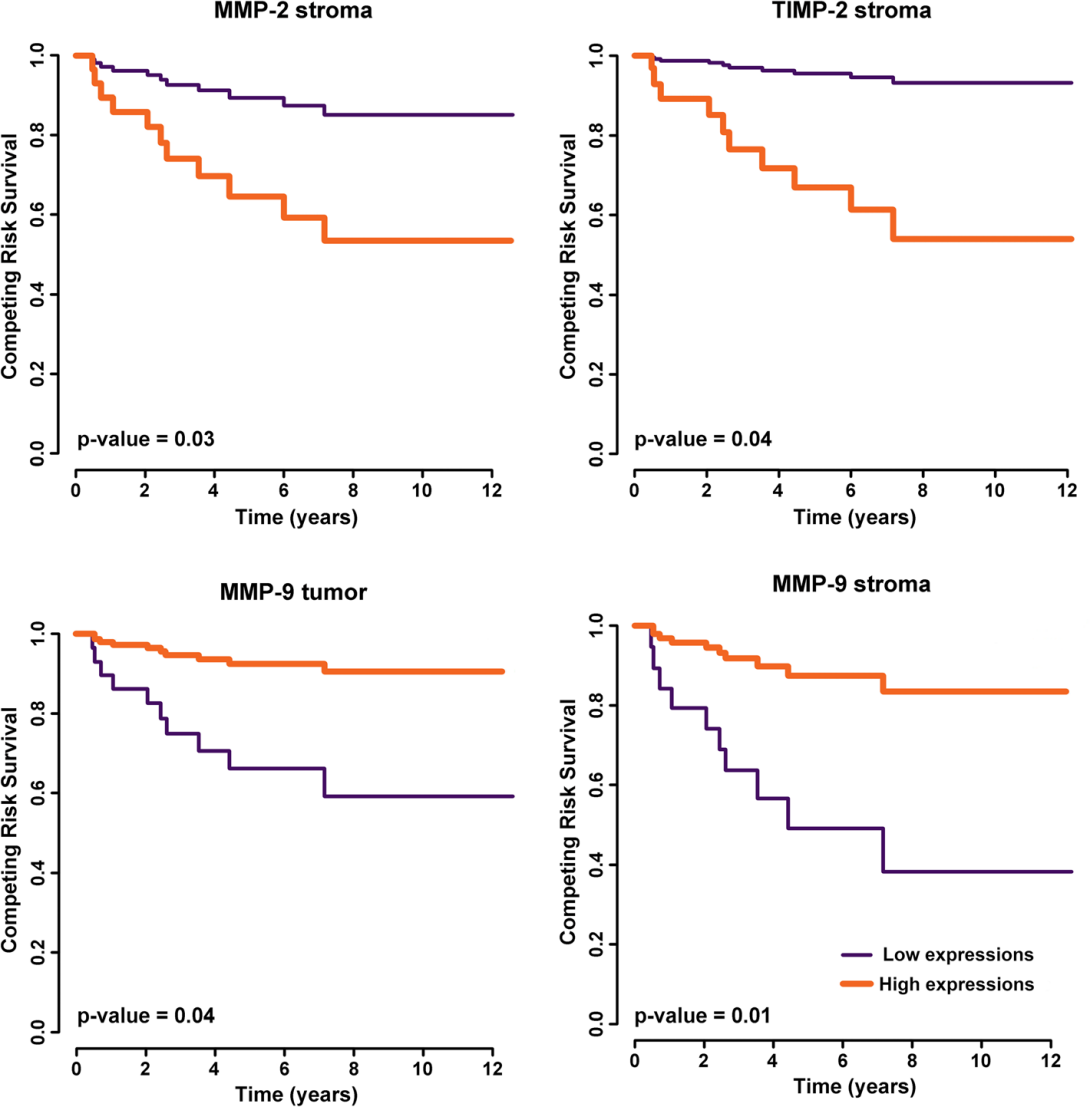
Competing risk survival curves of cervical cancer patients with high and low expression of stromal MMP-2, TIMP-2, MMP-9 and tumoral MMP-9

The median follow-up was 5.6 months. The last event occurred at 7.17 years, and the follow-up continued up to 12 years. The patients with high expression of MMP-2 and TIMP-2 in the stroma showed significantly poorer survival than those with low expression (*p* = 0.03; *p* = 0.04). MMP-9 expression, otherwise, showed an opposite association because of its protective association with cause-specific death. Cases showing low expression of MMP-9 in the tumor and stroma revealed higher survival rates (p = 0.04; *p* = 0.01) (Fig. 4).

## Discussion

High expression levels of MMP-2 and TIMP-2 in the stroma were associated with lower survival. Conversely, high expression of MMP-9 in the stroma and the tumor was associated with higher survival rates than low expression. The fact that associations between prognosis and stromal factors were more frequent than associations between prognosis and tumoral factors caught our attention. To discuss these results, we sought to better understand the role of stroma in the tumor microenvironment.

In 1889, Paget [27] hypothesized that cancer development could be dependent on its surroundings just like a seed depends on the soil. He was credited with being the first to postulate the importance of the microenvironment in tumor progression and metastasis and suggested that stroma and tumor need to be related to each other [28].

The stroma is the supportive and connective tissue, so when the epithelium changes, it changes as well. It is composed of many fibroblasts, vascular, lymphovascular, endothelial, and immune cells. Fibroblasts synthesize extracellular matrix (ECM) proteins, but in cancer, they also secrete other kinds of proteins, mainly MMPs, that degrade the ECM [29]. This ECM remodelling in the tumor microenvironment might allow the passage of tumor cells through the barrier of the host tissue and the migration of endothelial cells into the matrix. This can also induce neovascularization and release growth factors that enhance the process of tumor growth and invasion [30–34]. In addition, stromal cells can incite phenotypic and genomic changes in epithelial cells, even without a pre-existing tumor [35].

Sato et al. (2004) demonstrated that tumor cells in contact with peripheral stromal cells increase MMP activation, resulting in cancer progression [36]. In recent studies, a high proportion of stroma, indicated by a low tumor-stroma ratio, was identified as an independent prognostic factor that predicts a poor prognosis in colon, gastric and breast cancers [37–40]. These studies demonstrate the importance of analyzing the tumor along with its stromal tissue.

MMP-2 overexpression seems to be associated with poor prognosis in several types of gastrointestinal [41, 42] and gynecological cancers. A meta-analysis, that did not discriminate stromal and tumoral cells, found that high MMP-2 expression was associated with advanced stage, tumor invasion and metastasis in endometrial cancer [43], and MMP-2 was also shown to correlate with shortened survival in patients with breast carcinoma [44]. In our study, MMP-2 expression, both tumoral and stromal, was associated with cervical cancer-related death. Although the tumoral MMP-2 relative risk and survival curves could not be evaluated, its significance cannot be completely excluded among the other significant associations. We believe that if our sample size was larger, we could have cases of low expressions and the possibility to estimate the relative risk.

Regarding cervical cancer progression, Fernandes et al. (2008) found a progressive enhancement of MMP-2 expression in stromal cells from CIN III to cervical invasive carcinoma [45], possibly due to an imbalance with its main inhibitor, TIMP-2. TIMP-2 has two ideally opposite functions in the tissue microenvironment. They can directly bind to MMP-2 for inhibition, and they have a proteolytic role when they form a complex with MMP-14 and proMMP-2 releasing an active MMP-2 [46, 47]. Therefore, changes in TIMP-2 and MMP-2 expression can determine whether TIMP-2 can promote or inhibit tumoral progression [48]. Although it is associated with a favorable prognosis in many types of cancer [49–53], it can be associated with poor prognosis in others [54]. This relates to our study, which shows that high expression of TIMP-2 in the stroma is associated with poor survival. Considering the balance mentioned, its overexpression may be correlated with MMP-2 overexpression.

MMP-14 is also associated with tumor progression. Some authors found it independently associated with lower overall survival [55, 56], but others affirmed that its association with tumor progression may be due to its role in MMP-2 activation [46, 47]. Chenard et al. (1999) found that MMP-14 was present in all invasive carcinomas tested and almost exclusively expressed in stromal cells, suggesting that MMP-2 activation may be a peri-fibroblastic event [57]. In this study, stromal MMP-14 showed borderline significance, so a larger number of patients could have shown that MMP-14, along with MMP-2 and TIMP-2 in the stroma, was significantly associated with lower survival. The sample size only allowed for the identification of great differences, so if MMP-14 is, in fact, only associated with MMP-2 activation in survival, its significance may have been diluted by our massive MMP-2 significant results.

MMP-2 and MMP-9, both gelatinases, have been associated with tumor progression and tumoral angiogenesis [58], but in our analyses, MMP-9 had a different pattern of association. Indeed, MMP-9 had a positive association with survival in the competing risk analysis both in tumoral and stromal cells: the higher its expression was, the higher the survival rate.

Nilsson et al. in 2006 supported the concept that MMPs may have an antitumor feature. Through microdialysis, they found increased endostatin and MMP-2 and MMP-9 levels in mice with breast cancer treated with tamoxifen, and inhibition of MMP-2 and MMP-9 resulted in a significant decrease in endostatin levels. They suggested that MMP-2 and MMP-9 might modulate endostatin generation and consequently affect angiogenesis [59]. Later, Bendrick et al. demonstrated that MMP-9, when injected directly into the tumor, could reduce tumor size in mice with breast cancer. Their hypothesis was also based on the fact that MMP-9 can induce the expression of antiangiogenic endostatins [60, 61].

In a more recent study, Leifler et al. (2013) injected an adenoviral gene transfer of MMP-9 into breast cancer tumors. This resulted in decreased tumor growth and angiogenesis. The discussion focused on MMP-9 being a potent and significant releaser of antiangiogenic endostatins and an inducer of massive neutrophil infiltration. They also studied TIMP-1 because it is the natural inhibitor of MMP-9, but similar to our results, it did not show significance. TIMP-1 did not affect tumor growth or the immune response [62].

VEGF-A, on the other hand, is a well-known mediator of tumor angiogenesis in many solid tumors. It has been used as an inhibitor of angiogenesis in persistent cervical cancer cases [63]. However, VEGF-A, especially in the tumor, showed borderline significance with competing risk survival in our study. Once more, we believe that a larger sample size could reveal a significant association, especially because MMP-2 in tumors had such a high association with death from the disease, which could interfere with the analysis of these data.

The first strength of this study is the separation of the analysis of stromal cells from that of tumoral cells, which provided a better understanding of cervical tumor microenvironmental dynamics. Second, the most relevant MMPs and TIMPs were associated with tumor invasion and metastasis. As discussed, the small sample size was a weakness of the study because the findings of borderline significance could represent a true difference. However, the strongest associations with cervical cancer prognosis were successfully revealed, and therefore, these proteins could be considered prognostic factors and possible targeted therapies.

## Conclusions

In conclusion, high expression levels of MMP-2 and TIMP-2 in the stroma are significantly associated with poor survival in cervical cancer patients. MMP-9 expression is associated with a favorable prognosis. Clinically, these proteins can be used as prognostic factors and may guide therapeutic decisions so that women showing a poor prognosis can receive a more effective primary treatment.

## Data Availability

The datasets used and analyzed during the current study are available from the corresponding author upon reasonable request.

## Abbreviations

BM: Basement membrane
CAISM: Women’s Hospital Prof. Dr. Jose Aristodemo Pinotti
CC: Cervical cancer
95%CI: 95% confidence interval
CIN: Cervical intraepithelial neoplasia
CRS: Competing risk survival
ECM: Extracellular matrix
FIGO: Federation International of Gynecology and Obstetrics
HPV: Human papillomavirus
HR: Hazard Ratio
ICD: Classification of Diseases and Related Health Problems
MMP: Matrix metalloproteinase
mRNA: Messenger ribonucleic acid
TIMP: Tissue inhibitor of metalloproteinase
ROC: Receiver Operating Characteristic
VEGF: Vascular endothelial growth factor
UNICAMP: State University of Campinas
